# Multiobjective optimization algorithm for accurate MADYMO reconstruction of vehicle-pedestrian accidents

**DOI:** 10.3389/fbioe.2022.1032621

**Published:** 2022-12-05

**Authors:** Donghua Zou, Ying Fan, Ningguo Liu, Jianhua Zhang, Dikun Liu, Qingfeng Liu, Zhengdong Li, Jinming Wang, Jiang Huang

**Affiliations:** ^1^ School of Forensic Medicine, Guizhou Medical University, Guiyang, China; ^2^ Shanghai Key Laboratory of Forensic Medicine, Shanghai Forensic Service Platform, Academy of Forensic Science, Ministry of Justice, Shanghai, China

**Keywords:** traffic accident, accident reconstruction, multibody simulation, pedestrian injury, multiobjective optimization algorithm

## Abstract

In vehicle–pedestrian accidents, the preimpact conditions of pedestrians and vehicles are frequently uncertain. The incident data for a crash, such as vehicle deformation, injury of the victim, distance of initial position and rest position of accident participants, are useful for verification in MAthematical DYnamic MOdels (MADYMO) simulations. The purpose of this study is to explore the use of an improved optimization algorithm combined with MADYMO multibody simulations and crash data to conduct accurate reconstructions of vehicle–pedestrian accidents. The objective function of the optimization problem was defined as the Euclidean distance between the known vehicle, human and ground contact points, and multiobjective optimization algorithms were employed to obtain the local minima of the objective function. Three common multiobjective optimization algorithms—nondominated sorting genetic algorithm-II (NSGA-II), neighbourhood cultivation genetic algorithm (NCGA), and multiobjective particle swarm optimization (MOPSO)—were compared. The effect of the number of objective functions, the choice of different objective functions and the optimal number of iterations were also considered. The final reconstructed results were compared with the process of a real accident. Based on the results of the reconstruction of a real-world accident, the present study indicated that NSGA-II had better convergence and generated more noninferior solutions and better final solutions than NCGA and MOPSO. In addition, when all vehicle-pedestrian-ground contacts were considered, the results showed a better match in terms of kinematic response. NSGA-II converged within 100 generations. This study indicated that multibody simulations coupled with optimization algorithms can be used to accurately reconstruct vehicle-pedestrian collisions.

## 1 Introduction

The Global Status Report on Road Safety released by the World Health Organization indicates that approximately 1.35 million people die in road traffic crashes each year (WHO, 2018). Traffic accidents are one of the main causes of death in China, and pedestrians are an extremely vulnerable group during vehicle impacts. Once an accident occurs, there are disputes regarding responsibility for the accident and compensation for vehicle deformation and personal injury. Traffic police must identify the responsible party or parties to resolve these disputes. The traditional forensic methods that determine the process of an accident are mainly based on traces of evidence at the scene, vehicle deformations and injuries of the victims. Such empirical inference is subjective and sometimes controversial.

In recent years, multibody (MB) models and numerous other methods have become prevalent for reconstructing traffic accidents. J.R. Elliott et al. (Elliott et al., 2012) used the MAthematical DYnamic MOdels (MDAYMO) MB pedestrian model to quantitatively analyse the influences of vehicle speed, pedestrian speed and pedestrian gait on the transverse translation of the pedestrian’s head, head rotation about the vertical head axis and head impact velocity. Zou et al. (2019) reconstructed the kinematics of a scooter-microvan accident involving three riders by using MADYMO MB simulation software and explored the differences in injury risks and characteristics of scooter drivers and passengers, succeeding in identifying the driver as the party responsible for the accident. To investigate the characteristics of pedestrian head-vehicle contact boundary conditions and risk of pedestrian head injury as functions of kinematic-based criteria and lower extremity injuries, previous studies (Li et al., 2021a; Panday et al., 2021) have reconstructed many collision cases and applied MB modelling to reconstruct pedestrian kinematics in real-word collisions. Previous studies show that numerical simulation methods can be used to quickly and objectively reconstruct the accident process.

The process of reconstruction is a process of reduction to the best hypothesis, which requires many iterative studies to establish the most likely crash configuration. Although numerical simulations have been increasingly performed, the most common approach is still the trial-and-error method. Such a method has low efficiency and is greatly affected by analysts. In addition, the preimpact parameters and traces of crashes are not comprehensively analysed. The key challenges to applying simulation methods to reconstruct traffic accidents are constructing human-vehicle simulation models that resemble the real accident and obtaining accurate initial collision parameters while preventing the influence of subjective human factors. Our previous study (Sun et al., 2017) developed an improved method for using MADYMO MB simulation software and an optimization method (genetic algorithm) to reconstruct a real vehicle–bicycle accident. However, the input conditions and parameters for optimizing the simulation process by using algorithms were not fully considered. The optimization efficiency of different algorithms is another issue.

To ensure the accuracy of accident reconstruction, it is necessary to investigate the optimal design of initial collision parameters, which should simultaneously consider multiple parameter factors. We need to construct a multiobjective optimial design of the established accident model. The multiobjective optimization method shows its advantages in the processing of many problems in military applications, the automobile, ship, and aerospace industries and other fields (Ishida et al., 2011; Dai et al., 2019; Gobeyn and Goethals, 2019; Dong et al., 2021; Shirazi et al., 2021). Presently, the most popular multiobjective genetic algorithms are the Nondominated Sorting Genetic Algorithm II (NSGA-II) (Deb et al., 2002), Neighbourhood Cultivation Genetic Algorithm (NCGA) (Watanabe et al., 2002) and Multi-Objective Particle Swarm Optimization (MOPSO) algorithm (Mostaghim and Teich, 2003). NSGA-II and NCGA are nonnormalized methods that are commonly utilized in multiobjective optimization design. These multiobjective genetic algorithms can simultaneously optimize several objective functions while maintaining the diversity of the solutions. MOPSO (Feng et al., 2013), which was proposed in 2004, applies particle swarm optimization (PSO), which can be applied only for a single object to multiple targets and has become a controversial topic in the modern optimization field.

Because of the shortcomings of previous human-vehicle collision reconstruction methods and the continuous development of optimization methods, this study compares the optimization effects of three multiobjective algorithms on the multi-rigid-body simulation results for traffic accidents and conducts optimization design research on the initial parameters of a the collision. In this way, the conditions for the application of different algorithms can be obtained, and the accuracy of accident reconstruction can be improved.

## 2 Methods and materials

### 2.1 Optimization methods for accident reconstruction

Traffic accident reconstruction can be represented using known accident data, such as the braking distance of the vehicle, the distance the pedestrian was thrown, the deformation of the vehicle, the injury of the human body, the pedestrian posture and motion at the time of impact, and videos and data from attached active safety equipment, to reconstruct the accident process. Thus, the reconstruction problem can be considered as a multiobjective mathematical optimization problem, which is expressed in standard form:

$$\mathrm{Min}\;F\left(x\right)=F\left\{f_{1}\left(x\right),f_{2}\left(x\right),\cdots ,f_{n}\left(x\right)\right\},$$ (1)

$$f\left(x\right)=d_{marker}|t_{marker},a_{j}\leq x\leq b_{j},\;j=1,2,\cdots ,k,$$ (2)

$$C_{i}\leq g_{i}\left(x\right)\leq e_{i},\;i=1,2,\cdots ,k,$$ (3)

where *F* ( x ), *f* ( x ) is the objective function and subobjectives identify the quantities to be minimized; a_j_ and b_*j*_ represent the upper limit and lower limit of the optimization variable x , respectively; and d_marker_ is the relative Euclidean distance between the human marker and markers on the vehicle and the ground at the time of impact t_marker_. The functions *g*_*i*_ ( x ) are constraint functions that can be used to define the ranges of the design constraints.

**FIGURE 1:**
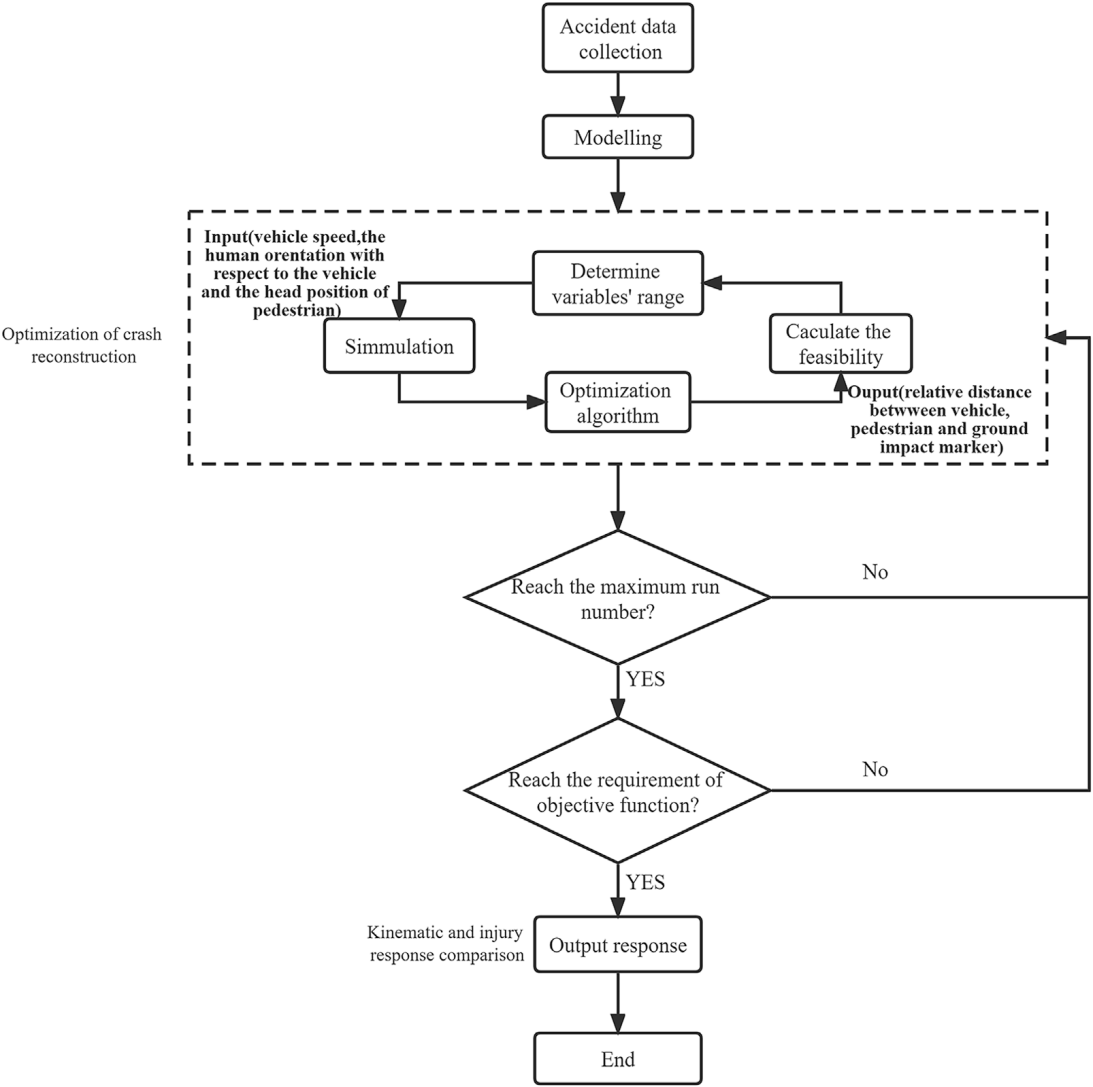
Workflow of traffic accident reconstruction and optimization.

The workflow of accident reconstruction optimization is shown in Figure 1. First, MB and facet models were built based on accident data. Second, preimpact parameters that affected the accident results, such as vehicle speed, human orientation with respect to the vehicle and head position of the pedestrian, were treated as initial design variables. Last, multiobjective genetic algorithms, such as NSGA-II, NCGA, MOPSO, were used to obtain optimal solutions. Each simulation of the multi-rigid-body model was set to terminate at 1.8 s.

### 2.2 Typical accident reconstruction

#### 2.2.1 Case report

**FIGURE 2:**
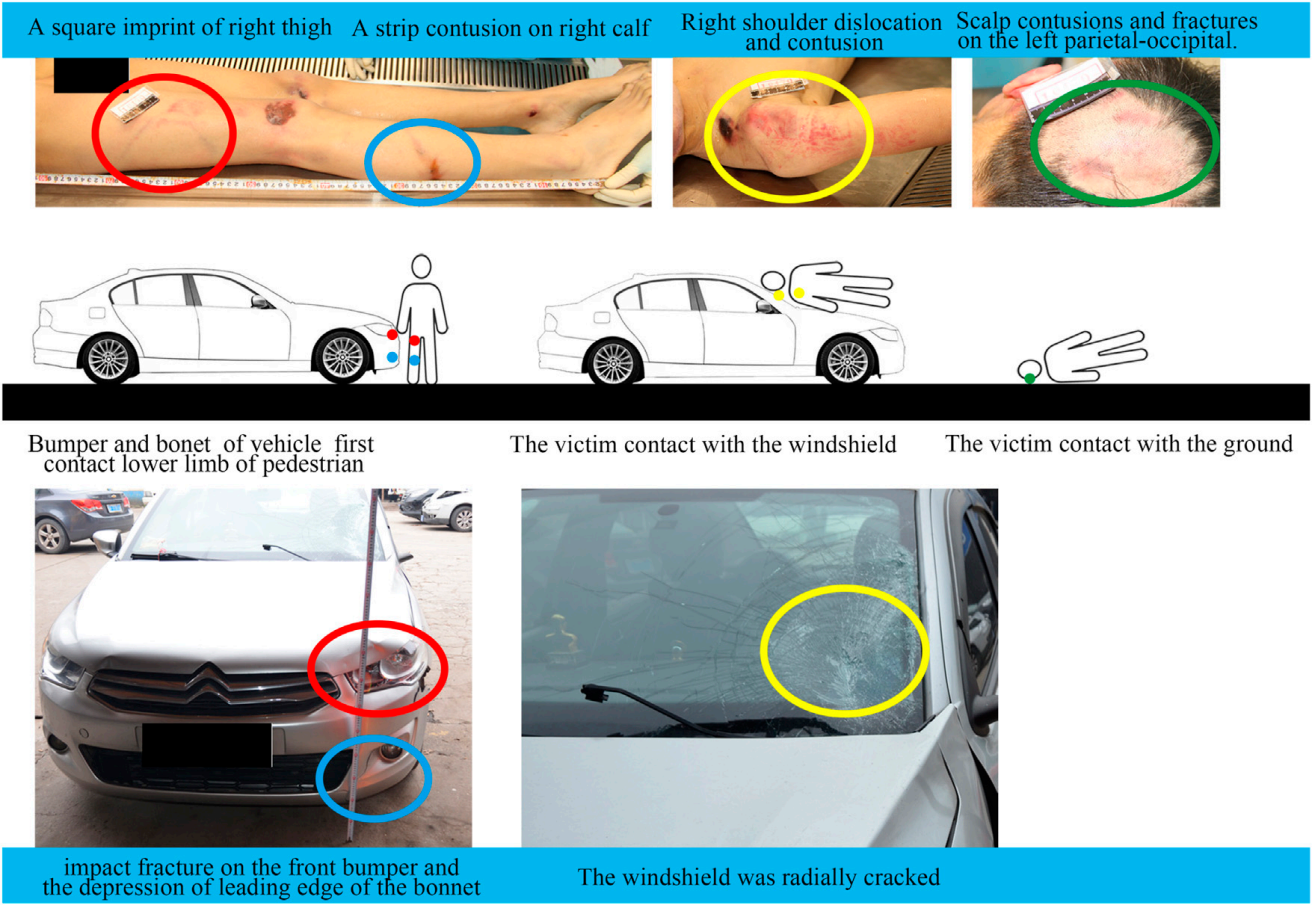
Pedestrian injuries and vehicle damages in different stages after vehicle-pedestrian traffic accidents. The contact between the pedestrian’s right thigh and the bumper cover is indicated in red, the contact between the pedestrian’s calf and the bumper is indicated in blue, the contact between the pedestrian’s shoulder joint and the windshield is indicated in yellow, and green indicates the first contact between the pedestrian’s head and the ground.

Data required for accident reconstruction were provided by the Academy of Forensic Science in Shanghai, including detailed documents about police investigations, witness testimonies, litigant statements, and a video record. The postmortem examination and vehicle inspection were performed by the Academy of Forensic Science. This study was approved by the Ethical Committee of the Academy of Forensic Science. All experiments were performed in accordance with relevant guidelines and regulations, and informed consent was obtained from the families of the deceased. There was a video from the roadside recording of the entire collision. The frame rate of the video was 16 frames per second, and the video resolution was 1,280 × 720 pixels. The accident is described as follows. At 7:00 p.m., an 18-year-old male was crossing a highway from north to south when he was struck by a Citroen vehicle moving from the west to the east on the highway. The cause of death of the pedestrian was a severe traumatic brain injury. The main injuries suffered by the victim were documented as follows (Figure 2):

- Scalp contusions and fractures on the left parietal-occipital
- Right shoulder dislocation and bruises
- Fracture of the lower part of the right femur, a square imprint bruise on the right thigh and a strip contusion on the right calf
- Numerous abrasions on other parts of the body

Deformations of the involved vehicle were mainly concentrated on its front left side. The lower left side of the windshield was radially cracked. The paint was scuffed, and there was an impact fracture on the lower left side of the front bumper. Depressions were observed at the leading edge of the bonnet (Figure 2).

#### 2.2.2 On-site reconstruction by UAV aerial photography

We selected the commercial DJI Inspire 2 (DJI, China) to document the accident scene in three steps. First, we calibrated the unmanned aerial vehicle (UAV) positioning system and camera if it had undergone long-distance transportation. Second, we planned the aerial photography route according to the site conditions and battery reserve. The flight height over the general accident scene was 30 m so that the drone could take high-resolution photographs while avoiding obstacles. The horizontal and vertical aerial image overlap rate was 80%. Third, we imported the aerial image into Context Capture software (Bentley, United States) to complete the construction of a 3D geometric model.

#### 2.2.3 Modelling

The MADYMO 50th percentile male model developed by Netherlands Organization (TNO), which consisted of 52 rigid bodies and had an outer surface described by 64 ellipsoids and two planes, was chosen as the human model. The model was developed and validated and was determined to satisfy the available biofidelity requirements in terms of kinematics, impactor forces, and accelerations in several body parts (Happee et al., 1998; Happee et al., 2000; Coley et al., 2001). Numerous attempts to use the 50th percentile model in reconstructions of real collisions showed that the model accurately predicted the global kinematics and impact points on the vehicle (TASS, 2013). To match the height and weight of the accident victim, we used the Generator of Body Data (GEBOD) method to scale the human model. The posture of the pedestrian was adjusted according to the previous frame of the video when the vehicle hit the pedestrian. We adjusted the hinge angles of the shoulder, elbow, hip, knee, ankle and head-neck joints so that the posture of the human model matched the posture of the pedestrian in the accident (arms bent inserted in the pockets, right leg in front and left leg behind in a walking gait, and body leaning slightly to the left).

**FIGURE 3:**
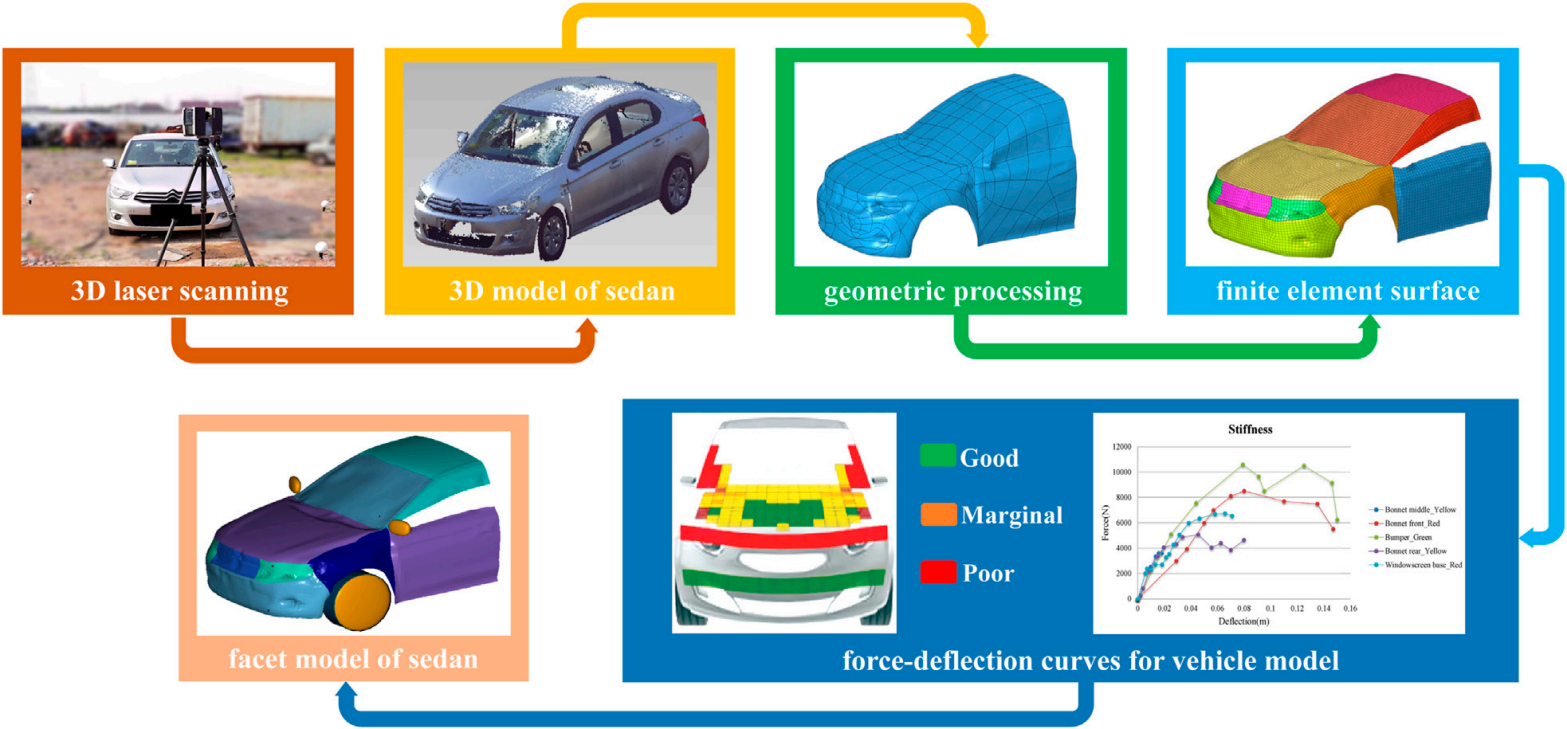
Process of reverse reconstruction of the vehicle model.

Faro Focus 3D S120 laser scanning with an accuracy range of 2 mm and postprocessing software FARO SCENE (FARO, United States) were used to scan the accident vehicle and obtain point cloud data that were processed in Geomagic 2017 software (3D Systems Corporation, America). The point cloud model was encapsulated to obtain a polyhedral model. Then, Hypermesh 2019 software (Altair Engineering Inc., America) was employed to process a mesh and form a finite element surface model of the vehicle. The node and unit data in the obtained K file of the finite element surface model were imported into the vehicle file built in MADYMO, and a facet model of the vehicle was obtained. This method ensured the accuracy of the geometry of the model vehicle. The European New Car Assessment Programme (EuroNCAP) database (Martinez et al., 2007) divided the front structure of a vehicle into three zones based on its aggressiveness against pedestrians. The contact stiffness characteristics of the front part of the model vehicle were selected based on structural stiffness test results from EuroNCAP for similar vehicles. One such matrix model of the crash reconstruction is shown in Figure 3. The simplified force deflection data for the bumper area were derived from the legform test, while the bonnet front area was obtained from the upper legform tests, and the bonnet middle area, bonnet rear area and windscreen were derived from the headform test.

#### 2.2.4 Prediction of impact speed based on the video

**FIGURE 4:**
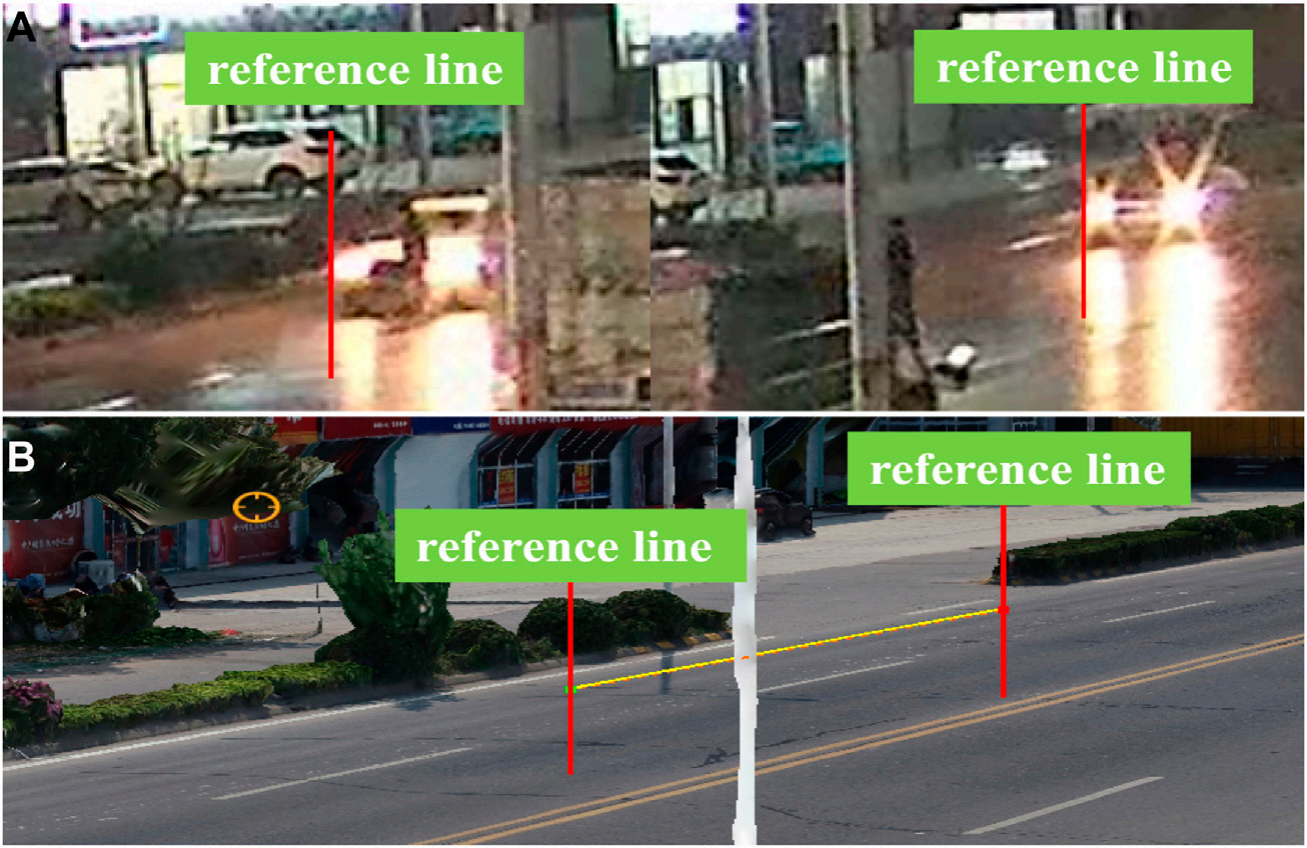
Measurement and calculation of vehicle collision speed. **(A)** Screenshot of surveillance video along the road (times interval: 16 frames). **(B)** 3D documentation of the incident scene by the UAV.

We played and analysed the continuous surveillance video using VOIT software (Hikvision, China). The virtual location method (Jiao et al., 2018) was utilized to match the location of the vehicle in the video with the scene of scanning reconstruction, and the reference lines were set to calculate the speed of the vehicle before the collision (Figure 4). The following contents were displayed in sequence according to the playing time; the video frame rate was 16 frames per second. According to Figure 4, before the collision, the reference distance between the vehicle in the 1st frame and the vehicle in the 17th frame was approximately 17.96 m, which was measured with the 3D model of the accident scene by UAV photography. The following formula (Feng et al., 2018) was used to calculate the vehicle speed, and the known parameters were substituted into the formula to calculate the vehicle speed in the human-vehicle collision. This velocity was a preliminary estimate. Therefore, we set the range of the velocity variable to [16 m/s and 20 m/s].

$$v=\frac{1}{t}\approx \frac{17.96}{\left(17-1\right)\times \left(\frac{1}{16}\right)}=17.96m/s.$$ (4)

#### 2.2.5 Setting of the friction coefficient

In this case, the accident occurred on a rainy day and on wet asphalt pavement. After the collision, the pedestrian was thrown onto the ground on the left side of the vehicle. The friction coefficient between the wheels and the ground in this study was obtained by substituting the regression formula in the literature (Zheng et al., 2011) according to the tire condition, the material of the road surface, the temperature of the road surface and the humidity. The friction coefficients between the pedestrian and the ground, and between the pedestrian and the vehicle were also obtained from the literature (Mondal et al., 2016). The friction coefficients between the vehicle and the ground, between the pedestrian and the ground, and between the pedestrian and the vehicle were respectively 0.55, 0.6, and 0.3, respectively.

### 2.3 Determine the range of the optimization variables

**FIGURE 5:**
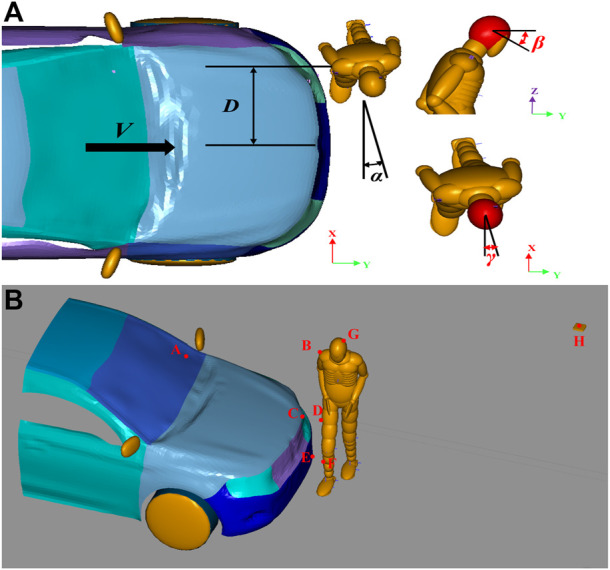
Schematic of the optimization model **(A)** Different initial collision parameters (optimization variables) **(B)** Positions of the four collision marker point pairs.

Five optimization variables were set in the optimizations according to a previous study concerning accident reconstruction (Wang et al., 2021a), as shown in Figure 5A, including the initial speed of the vehicle-pedestrian collision (V), the distance of the pedestrian relative to the long axis of the vehicle (D), the angle of the pedestrian relative to the collision vehicle (α), the angle of the human head posture turning up-down (β) and the angle of left-right rotation (γ). The range of optimization variables and their initial values determined according to the posture of the subjects in the video are shown in Table 1.

**TABLE 1:** Range of different initial collision parameters and the initial value setting of the optimization model.

Optimization parameters	Minimum	Maximum	Initial values
*V*/(m/s)	16.00	20.00	18
*D*/m	0.14	0.54	0.34
*α*/rad	0.30	0.70	0.5
*β*/rad	−0.20	0.20	0
*γ*/rad	−0.20	0.20	0

### 2.4 Design of experiments

Different preimpact parameters lead to different accident processes and personal injuries. According to inspections of the human body and vehicle and a survey of the site, we selected the characteristic injuries on the corpse to match the deformation of the vehicle and trace of the site. We set up four pairs of markers, including three pairs of contact points between the vehicle and the pedestrian, and one pair of contact points between the pedestrian and the ground. The locations of the four groups of collision points are listed in Table 2. The four subobjectives represent the relative distances between each group of markers. The objective function was the sum of the four subobjectives. In each optimization design, constraints of the distance between each group of collision markers were set from 0 m to 2 m. We assumed that distances beyond these constraints did not correspond to reality and that when the objective function approached a minimum, the reconstruction resembled the real accident. The initial relative positions of the pedestrian and vehicle were adjusted based on video data. The final numerical model is shown in Figure 5B.

**TABLE 2:** Setting of four pairs of collision marker points and objective function.

Group	I	II	III	IV
Collision markers	A: the center of the spiderweb fracture pattern of the windshield. B: the right shoulder of the pedestrian	C: the bumper area near the left headlight. D: the square mark on the middle of the right thigh	E: the lower left front of the bumper. F: the outer middle of the right calf	G: where the human head hits the ground after a collision. H: the position of contact with the ground in the video
subobjectives	*d* (AB)	*d* (CD)	*d* (EF)	*d* (GH)
Objective function	*d* (objective) = *d* (AB)+*d* (CD)+*d* (EF)+*d* (GH)			

The purpose of this study was to explore how to use an improved optimization algorithm combined with MADYMO MB simulations and crash data to accurately reconstruct vehicle–pedestrian accidents. Traffic accidents are such complex events that we cannot acquire complete accident data. Thus, the key challenges were determining how the objective functions affected the reconstruction results and which multiobjective optimization algorithm achieved better performance. Three widely utilized algorithms—NSGA-II, NCGA, and MOPSO algorithm—were considered for the accident reconstruction. Theoretically, as more collision contact marker pairs were employed for matching, the more complete the reconstructed information matched to the accident and the better the reconstruction. Based on the established human-vehicle collision model, four subobjectives were used to investigate which algorithm provided better performance for accident reconstruction optimization. In addition, 11 optimization groups were set according to the number of subobjectives and different contact marker combinations, as shown in Table 3. Among the above three algorithms, the algorithm with the best performance was selected to optimize the 11 optimization groups.

**TABLE 3:** Optimization groups based on the number of collision marker pairs and the type of collision contact. There are 11 groups based on the number of subobjectives and the contact during the collision.

The number of subobjectives	Vehicle-pedestrian contact	Vehicle-pedestrian-ground contact
2	I and II	I and IV
	I and III	II and IV
	II and III	III and IV
3	I, II and III	I, II and IV
		I, III and IV
		II, III and IV
4	—	I, II, III and IV

### 2.5 Simulation

In the optimization simulation, all algorithms maintained the same maximum run sizes. For NSGA-II and NCGA, the size of the population was 20, and the number of generations was 20. For MOPSO, the maximum number of iterations was 20, and the number of particles was 20. In total, 400 simulations of each group were performed in workstations (Intel Xeon E5-2690 central processing unit (CPU) with 8 cores and 64GRAM). Notably, the setting of simulation times was based on empirical experiments. Theoretically, the larger the number of simulations is the larger the number of iterations and comparisons to obtain the optimal solution. The above three algorithms were executed using Isight 2017 software (DS SIMULIA). The computation time lasted approximately 100 h for each group. In the MADYMO simulation, we used the modified Euler method of MADYMO with a time step of 2.0^–5^ s × 10^–5^ s. The CPU duration for each analysis was set to 1.5 s. The real time needed for each simulation was approximately 15 min. The fracturing leg model option was selected.

### 2.6 Validation

The purpose of this study was to establish a method for accurately reconstructing the accident process while comparing the effects of multiple algorithms and different collision parameters on the optimization results. To further investigate the applicability of this method, it was necessary to validate the results. To verify the correctness of the method in this study, two other vehicle-pedestrian accident cases were randomly selected for reconstruction to achieve verification. In these two cases, due to the rescue and road clearance after the accidents, we could not obtain sufficiently accurate locations of pedestrian landing points based on the accident data. Therefore, the marker points in both cases were set at the contact position of the pedestrian’s head with the windshield and the contact position of the pedestrian’s femur with the front cover of the vehicle bonnet. Three different optimization algorithms were employed, and the optimization results were compared with the research findings and with the accident consequences to verify the accuracy of the reconstruction.

## 3 Results

### 3.1 Multi-rigid-body simulation results

**FIGURE 6:**
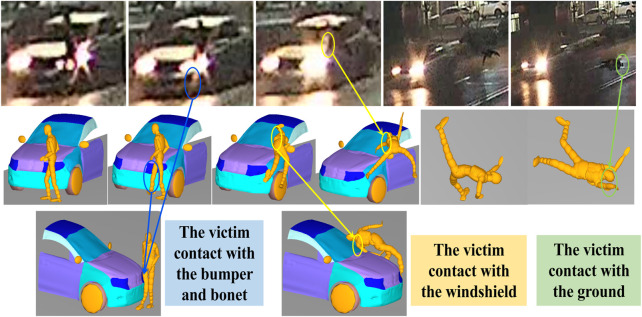
Comparison of kinematic response between simulation and accident (informed consent was obtained from the families of the deceased for the relevant content in this figure).

The reconstruction of the multi-rigid-body model provided dynamic visualization of contact between the pedestrian, vehicle and ground during the reconfigured accident and a comparison with the actual accident situation for vehicle deformations and human injuries. Using different optimization models and optimization methods, an optimal solution was obtained and reimported into MADYMO software for calculations to obtain the accident process that best described the actual accident. Figure 6 shows the relative positions of the vehicle and pedestrian in real life and the reconstructed accident. After the pedestrian was struck by the vehicle, the victim rotated in the direction opposite to the vehicle’s progress until the pedestrian’s right shoulder collided with the windshield of the vehicle. Next, the pedestrian continued to flip in the air and was thrown onto the vehicle on the left side of the road. In the reconstruction results, the position of contact between the pedestrian’s right shoulder and the shattered windshield of the vehicle, the position of impact between the pedestrian’s injured right leg and the vehicle’s bumper, and the position of impact between the pedestrian’s head and the ground matched the video data for the real accident, proving that the reconstruction was accurate. The simulation results showed that the value of HIC was 1882.9, predicting a 96% probability of AIS3 head injury according to relevant studies (Li and Zhu, 2009; Gao et al., 2020; Wang et al., 2021b; Wang et al., 2022a), which was consistent with the death of pedestrians due to severe craniocerebral injury in this accident. Relevant studies (Hu et al., 2020) showed that the range of forces leading to femur fracture was 3 kN–10 kN. In the present simulation, the maximum force on the lower femur was 10.093 kN (11.8 m), which was consistent with the pedestrian’s right lower thigh fracture caused by the vehicle bumper. Therefore, the predicted injury condition of the human body model was consistent with the actual accident. In these two validation cases, the validity of the reconstructions was evaluated by comparing the simulated pedestrian kinematics and the pedestrian injuries with the information obtained from accident investigations. The results suggest that these two accidents were well reconstructed.

### 3.2 Optimization results of different algorithms

All operations for each algorithm and each optimization group were automatically executed 400 times. The global optimum results for the different algorithms are shown in Table 4. The value of the objective function was the sum of the values of the different subobjectives, that is, the sum of the distances between pairs of corresponding collision marker points; therefore, the larger the value was, the worse the result. NSGA-II had the smallest objective function with a value of 0.1921, followed by NCGA with a value of 0.2064 and MOPSO with a relatively large objective function with a value of 0.2898. In the validation cases, similar results were obtained, as listed in the Appendix.

**TABLE 4:** List of the global optimal solution parameters of the group of “I, II, III and IV” optimized by different multiobjective optimization algorithms.

Different algorithms (I, II, III, and IV)	*V* (m/s)	*D* (m)	*α* (rad)	*β* (rad)	*γ* (rad)	*d* (AB) (m)	*d* (CD) (m)	*d* (EF) (m)	*d* (GH) (m)	*d* (objective) (m)
NSGA-II	17.3485	0.3186	0.6650	0.0298	−0.1895	0.1047	0.0493	0.0142	0.0239	0.1921
NCGA	16.5232	0.2889	0.6683	−0.1689	0.1185	0.1235	0.0396	0.0161	0.0271	0.2064
MOPSO	16.0000	0.2662	0.3565	−0.0547	0.0008	0.0612	0.0387	0.0111	0.1788	0.2898

**FIGURE 7:**
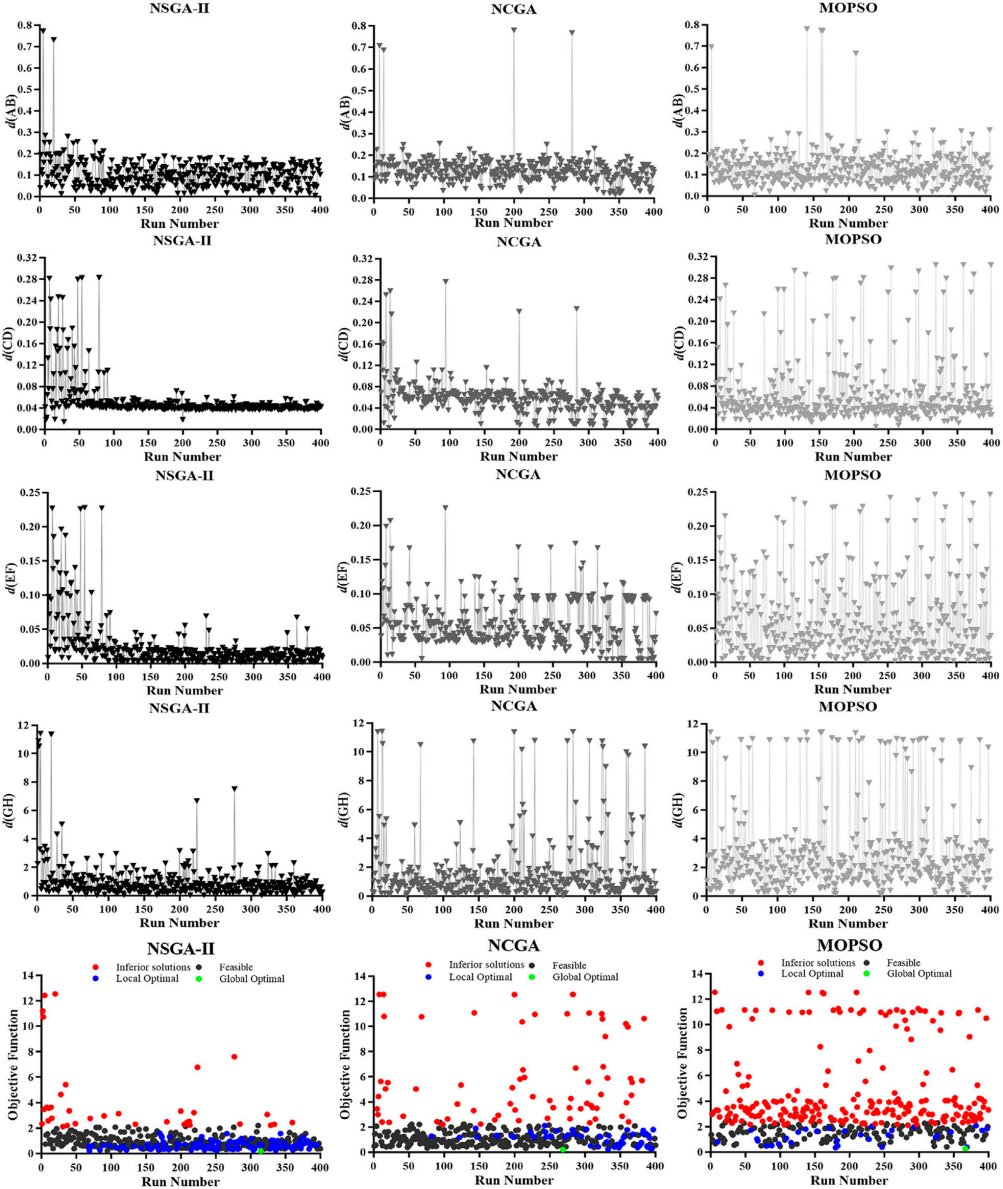
Simulation histories of different algorithms. The three columns from left to right are NSGA-II, NCGA, and MOPSO. The five rows from top to bottom are d (AB), *d* (CD), *d* (EF), *d* (GH), and *d* (objective). The *x*-axis of each Pareto graph indicates the number of runs, and the *y*-axis indicates the value of each sub objective function and the objective function.

The simulation histories of different algorithms are shown in Figure 7. The four subobjective functions significantly converged for NSGA-II, the first two subobjective functions (but not the last two) converged for NCGA, and none of the four subobjective functions converged for the MOPSO. The global optimal solutions for the three algorithms (NSGA-II, NCGA, MOPSO) occurred at the 315th iteration, 269th iteration and 367th iteration, respectively. Fewer inferior solutions (35) and more local optimal solutions (99) were obtained for NSGA-II compared to the NCGA (70 and 55, respectively) and compared to MOPSO(224 and 31, respectively). All the noninferior solutions were concentrated in the range from 0 m to 2 m, and all of the inferior solutions were greater than 2 m. The convergence of the optimization results for the validation cases showed similar results. In the two validation cases, it is also evident that the performed simulations have the same tendency.

**FIGURE 8:**
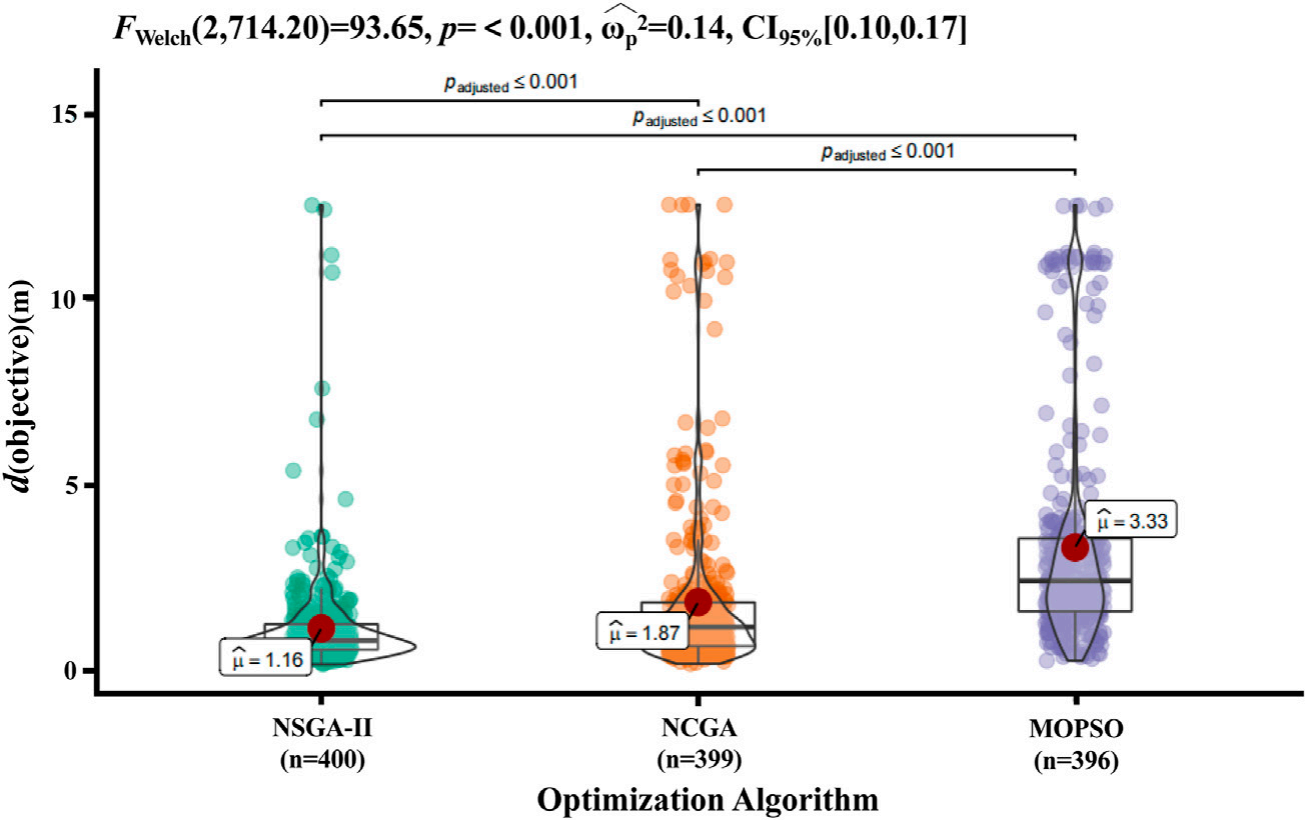
Objective function distribution of different multiobjective optimization algorithms. Data were analysed by one-way ANOVA, and the results were considered statistically significant when *p* < 0.05. The red dots indicate the average value of the objective function for each algorithm after removing the outliers.

One-way ANOVA was conducted to evaluate the distribution differences among the results of the data of the three different algorithms for the same group. As shown in Figure 8, with the same optimization parameters, the three algorithms had significantly different effects on the optimization of accident reconstruction. After removing outliers, the optimal results of different algorithms were significantly different (F = 93.65 and *p* < 0.001). The mean values of the objective function were 1.16 m, 1.87 m, and 3.33 m, and the median values were 0.830 m, 1.196 m, and 2.438 m for NSGA-II, NCGA, and MOPSO, respectively. The distribution of optimization results was more concentrated for NSGA-II than for NCGA, and the distribution was more discrete after optimization by MOPSO.

### 3.3 Optimization results of different preimpact parameters

As shown in Table 5, the optimal results for the different optimization groups showed that the achievable distance after optimization between model positions and observed positions was greater than 1 m, i.e., 1.7484 m, 1.0634 m, 1.7922 m, and 1.7417 m, when only vehicle-pedestrian contact was considered. However, all of the achieved distances after optimization between the model positions and the observed positions were less than 0.5 m, i.e., 0.1816 m, 0.3843 m, 0.3407 m, 0.1354 m, 0.2415 m, 0.2479 m, and 0.1921 m, when pedestrian-ground contact was simultaneously considered. When only the contact between the pedestrian and the vehicle was considered, the achieved distance after optimization between model positions and observed positions was significantly higher compared with the distance obtained when both the vehicle-pedestrian contact and pedestrian-ground contact were considered. This finding indicates that in addition to vehicle-pedestrian collision contact, the reconstruction combined with the information about the pedestrian landing point can effectively reduce the achievable distance after optimization between model positions and observed positions. When only the pedestrian-vehicle contact was considered or when the vehicle-pedestrian-ground contact was simultaneously considered, the distance was always at the same level for the same number of collision marker point pairs. This distance did not significantly decrease with the increase in the number of marker point pairs, but the minimum value appeared in group 8. In all simulations, the distance between the shoulder marker and the windscreen marker and that between the head marker and the ground considerably fluctuated, while the two groups of distances associated with the markers of the legs fluctuated less.

**TABLE 5:** List of the global optimal solution parameters of 11 optimization groups based on the number of collision marker pairs and collision contact types by NSGA-II.

Optimization groups (NSGA-II)	Groups	*V* (m/s)	*D* (m)	*α* (rad)	*β* (rad)	*γ* (rad)	*d* (AB) (m)	*d* (CD) (m)	*d* (EF) (m)	*d* (GH) (m)	*d* (objective) (m)
1	I and II	17.8211	0.2675	0.3741	−0.1441	0.1857	0.0033	0.0352	0.0098	1.7001	1.7484
2	I and III	17.2523	0.2760	0.3729	−0.1156	−0.1847	0.0098	0.0388	0.0031	1.0117	1.0634
3	II and III	17.0202	0.2772	0.3323	−0.0507	0.1261	0.0497	0.0369	0.0024	1.7032	1.7922
4	I, II and III	17.1558	0.2829	0.4149	−0.0284	−0.0753	0.0028	0.0400	0.0031	1.6958	1.7417
5	I and IV	16.0181	0.2527	0.4412	−0.1870	0.0760	0.0267	0.0500	0.0209	0.0839	0.1816
6	II and IV	16.9292	0.3662	0.4219	0.0042	0.0250	0.2017	0.1020	0.0654	0.0151	0.3843
7	III and IV	16.3664	0.3451	0.3633	0.0620	0.0736	0.1898	0.0834	0.0528	0.0148	0.3407
8	I, II and IV	16.5461	0.3061	0.6233	0.1572	0.1298	0.0564	0.0414	0.0049	0.0327	0.1354
9	I, III, and IV	16.4189	0.3063	0.4999	−0.0253	0.0854	0.0920	0.0439	0.0119	0.0937	0.2415
10	II, III, and IV	16.1749	0.2746	0.4315	−0.1880	0.0643	0.0694	0.0393	0.0115	0.1278	0.2479
11	I, II, III, and IV	17.3485	0.3186	0.6650	0.0298	−0.1895	0.1047	0.0493	0.0142	0.0239	0.1921

## 4 Discussion

### 4.1 Credibility and efficiency of the optimization method

The reconstruction of traffic accidents has always been essential for authorities to make impartial and informed decisions. Typically, a reconstruction of the dynamics may be requested in the event of traffic accidents, which helps authorities determine the responsible party in an accident. In recent times, bioengineering techniques have become increasingly involved in forensic disputes (Durante, 2019; Li et al., 2021b; Chen, 2021). The application of MB dynamics simulations for collision reconstruction may have significant advantages over traditional expert approaches in some cases. Expert assessment of the compatibility of the injuries, vehicle deformation and accident scenario is largely dependent on the theoretical and practical knowledge, while the use of simulation software allows this human factor to be minimized when an automatic optimization is involved. The simulation can provide more information, including information about the complete accident process, impact forces and preimpact conditions. The reconstruction results could be output as an animation, which can be useful for visualizing and describing the traffic accident scenario in a courtroom.

However, the uncertainty of reconstruction results is still an important issue. There are two general viewpoints on numerical simulation in traffic accident accidents. The first viewpoint is using uncertainty analysis methods to build an approximate model based on several simulation samples, such as the response surface model (RSM) method (Ming et al., 2014). This method is usually employed in conjunction with random sampling, such as Monte Carlo sampling and Latin hypercube sampling (Wang et al., 2022b). Zou et al. (2015) adopted PC-Crash to analyse the speed of an accident and to ascertain whether the driver was speeding based on extreme value theory and convex model theory. In addition, an improved probability-interval method was proposed for using probabilistic methods and interval trajectories to analyse the uncertainty of the reconstructed results of traffic accidents (Zou et al., 2017). The authors also proposed two improved experimental design methods, the first method is based on orthogonal design (OD) and is referred to as orthogonal design (OBD); and the second method is based on uniform design (UD) and is referred to as multiple response surface uniform design (MUD), which yields more accurate reconstruction results (Zou et al., 2018). This is an effective way to improve the reliability of an accident reconstruction result, especially in regard to predicting the range of collision speeds. However, not all variables and simulation results can be estimated from an approximate model. A best fit result is more appropriate when describing the location of the collision, posture of the victim, degree of injury, and dynamics of the whole accident process. To achieve the best-fitting results, analysts often adjust initial variables according to experience and intuition. Many simulations were needed when the results were unsatisfactory (Peng et al., 2013; Nie and Yang, 2014). Overall, the proposed optimization methods adopted the relative distances between pairs of contact points as optimization targets. The corresponding ranges of the initial variables were determined by existing accident data. For instance, we can estimate the range of vehicle collision speeds by surveillance video analysis. The value of a variable changes automatically in the optimization process. The injury outcomes and kinematics response can be validated by actual injuries and the existing traces. Therefore, the reconstruction result in our operational framework was the global optimal solution within a reasonable range of initial variables.

Another question is the accuracy of the models and accident data. The vehicle model is often constructed of ellipsoidal bodies of various sizes, thereby creating a certain gap between the model and the actual vehicles (Okamoto et al., 2003; Zou et al., 2019). By reconstructing the vehicle with 3D laser scans in this study, a multi-rigid-body model of the vehicle that was more accurate than the previous ellipsoid model was obtained. The height and weight of the mannequin were adjusted to match those of the accident victim. Reconstruction of the road at the scene by UAV photogrammetry also provided a more complete view of the accident road information and more accurate measurement. After optimization by NSGA-II, there was good consistency for the kinematic response of the pedestrian between the simulation and surveillance videos, and the injury analysis was consistent with the medical reports. The proposed approach is valid for highly improving reconstruction efficiency without simultaneously compromising accuracy, which provides more convincing evidence for forensic examinations and legal cases.

### 4.2 Effect of different optimization algorithms on optimization results

The purpose of this study was to achieve multiobjective optimization and to improve the accuracy and efficiency of accident reconstruction. The nature of optimization was to obtain a set of parameters that minimize the achievable distance between model positions and observed positions, so it is necessary to select an appropriate multiobjective algorithm. Multiobjective optimization could obtain a set of solutions where the decomposition of the assessment function into different objectives leaves room for more flexible solutions that cannot be reached with the single objective approach (Coello et al., 2007). Three widely employed multiobjective optimization algorithms were evaluated, among which NSGA-II has the best performance. The value of the global optimal solution for NSGA-II was the smallest, followed by NCGA and MOPSO. The objective function was the sum of the subobjective functions, and the closer the sum of the subobjective functions was to 0, the higher the accuracy of accident reconstruction and the better the collision results matched the actual accident. On the other hand, NSGA-II was optimized to yield the fewest inferior solutions and the most local optimal solutions, and therefore, was able to produce more alternative design solutions than the other two algorithms (Figure 7). Additionally, NSGA-II showed a better convergence in each subobjective function, and the local optimal solutions continued to increase and tended to be concentrated with the number of runs. The NSGA-II completed convergence within 100 generations (Untaroiu et al., 2009). In the two validation cases, it is also evident that the performed simulations have the same tendency. The powerful performance of NSGA-II was also confirmed by the study of Wang et al. (2020).

Figure 8 shows that after optimization of NSGA-II, the distribution of results was concentrated in a small region of values with relatively few discrete values, followed by NCGA, and that MOPSO had a larger distribution range, a large region of values in a large numerical area and more discrete values. Although the one-way ANOVA results showed significant differences between NSGA-II and NCGA, they had a similar performance in the convergence and the value of the global optimal solution. One reason may be that NSGA-II and NCGA are widely utilized genetic algorithms (GAs) that mimic heredity and evolution, which can effectively handle multiobjective and nonlinear problems with good exploratory properties. NSGA-II with an elitist strategy uses stochastic search methods based on the imitation of natural biological evolution. Its search mechanisms mainly included the preservation scheme of excellent solutions identified in the search, the assignment scheme of appropriate fitness values and a parameter-free sharing scheme. NCGA also includes neighbourhood crossover, but unlike the results of random selection, it can select individuals who were closer to each other in the crossover operation. Therefore, once there is a discrete value, there will also be a discrete value between adjacent generations. This finding may explain why NCGA converges faster but has more inferior solutions in the optimization process than NSGA-II. Both of algorithms try to achieve precise exploitation, and similar results have indicated that NSGA-II and NCGA had better performance for airbag design and optimization (Park, 2017). However, although MOPSO is similar to GA and can produce a population per generation, it does not have evolutionary processes such as selection, crossover and mutation (Shi, 1998). Therefore, MOPSO did not exhibit convergence and had the largest number of inferior solutions.

### 4.3 Effect of different preimpact parameters on the optimization results

**FIGURE 9:**
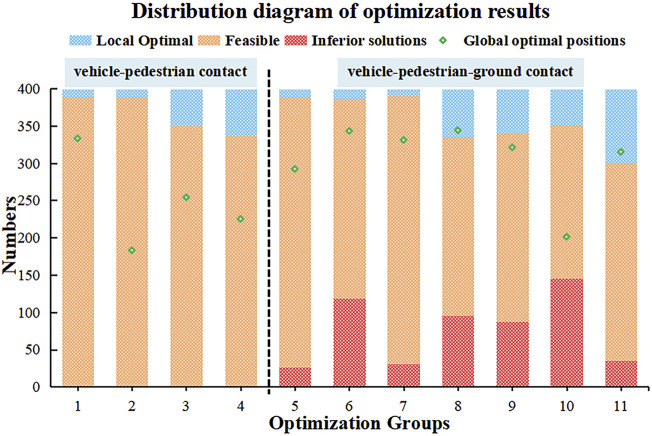
Distribution of the number of different quality solutions in the 11 optimization groups.

The multiobjective optimization algorithm can randomly value each design variable, and the generated test points can consider the influence of each variable on the collision results, thus ensuring the randomness and independence of the sample. To control for variables, we employed the same algorithm, size of the population and number of generations. Figure 9 shows the distribution of the 11 groups of optimization results after the NSGA-II analysis. Depending on the number of collision contact markers and subobjectives, the number of Pareto noninferior solutions and local solutions obtained after the optimization of each group varied, as did the location where the optimal solution appeared during the calculation. When only vehicle-pedestrian contact was considered, there were almost no inferior solutions. There was a certain number of inferior solutions when vehicle-pedestrian-ground contact was considered, which may be attributed to the greater uncertainty of the human body’s throw to the air in the landing stage after contact with the vehicle. In addition, the number of subobjectives involved in the optimization process varied, resulting in a different number of local optimal solutions. In general, as the number of subobjectives involved in the optimization process increased, the number of available alternatives increased. In addition, there was no obvious relationship between the position of the optimal solution and the number of subobjectives and collision contacts, but most of the optimal solutions were observed after 200 simulations. Therefore, the setting of simulation times needs to be taken seriously in future optimization designs.

**FIGURE 10:**
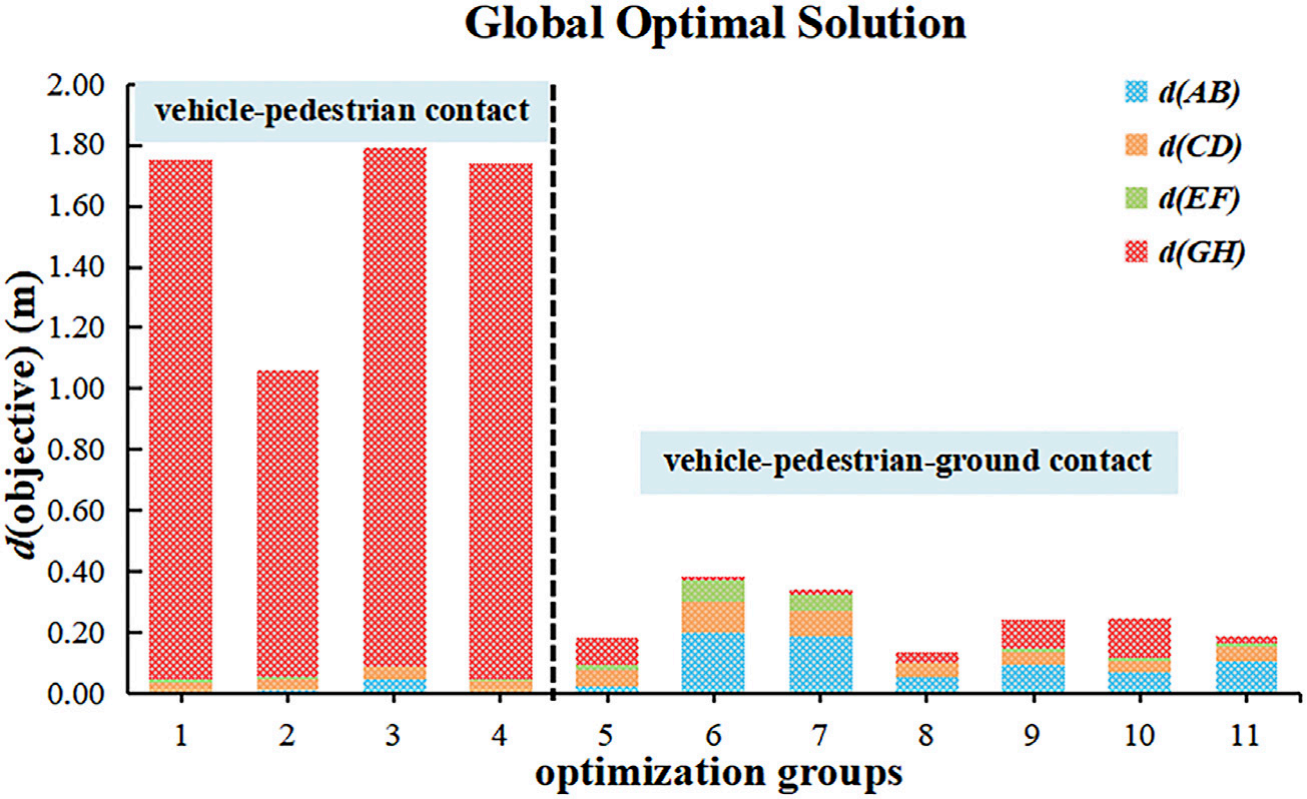
Achieved distances after optimization between model positions and observed positions of the global optimal solutions for the 11 optimization groups.

To be more persuasive, Figure 10 shows the distribution of the achieved distance after optimization between model positions and observed positions for the 11 optimization groups. The achieved distances after optimization between model positions and observed positions were generally larger when vehicle-pedestrian contact was considered than when vehicle-pedestrian-ground contact was considered. This finding shows that during the progress of traffic accident reconstruction and optimization, considering only vehicle-pedestrian collisions without considering collisions between the pedestrian and the ground led to inaccurate reconstruction results. Notably, the distance between the pedestrian’s right shoulder joint marker and the vehicle’s windshield marker fluctuated to some extent and was usually larger than the situation when only vehicle-pedestrian contact was considered. The distances between the other two collision marker pairs (pedestrian lower limb markers with the bumper marker and vehicle bonnet marker) were usually smaller, as these markers indicated the initial contact between the vehicle and the pedestrian. One plausible explanation is that leg injuries are often caused in the initial stages of an accident and therefore fluctuate less. For the results, it was clear that at least one pair of markers in each of the three postaccident phases (collision between vehicle and pedestrian, human body flipping in the air, and human body hitting the ground) was required for the accident reconstruction and optimization results and improved the accuracy of the accident reconstruction.

### 4.4 Representativeness of application cases and generalization of optimization methods

The proposed optimization methods adopted the relative distances between pairs of contact points as optimization targets based on the injuries, vehicle deformations and traces of the incident scenario. Injuries typical of vehicle-pedestrian crashes when the pedestrian is in a vertical position include contusions, abrasions, lacerations and fractures of the tibia, fibula and hip or pelvis, which are usually caused by the bumper or bonnet front of a vehicle; traumatic head injuries, including scalp lacerations and skull fractures occurring as a result of a contact with a windscreen and its surrounding frame; and injuries contacting the ground caused by pedestrians falling to the ground (Nogayeva et al., 2021). Craniocerebral injuries and lower limb injuries are the most common types of injuries sustained in pedestrian-vehicle accidents (Airaksinen et al., 2020). These injuries are often influenced by the speed of the vehicle during the collision, the angle of the impact with the pedestrian and the relative lateral positions of the pedestrian and vehicle. The angle of the head at the time of collision also affects the location and shape of a brain injury. Therefore, we applied the corresponding five parameters as optimization variables in the present case. These injuries also reflect the three stages of the process of a pedestrian being hit by a vehicle (Monfort and Mueller, 2020; Nogayeva et al., 2021). We often correspondingly observe relative vehicle deformation and contact traces. Hair, bloodstain, and clothing fibres also indicated the location of the contact point between the human body and the vehicle or ground. In the typical case presented in this paper, we created four pairs of collision markers based on the contact positions of bumper, bonnet front, windscreen and ground, which represented the common situation in vehicle-pedestrian traffic accidents. However, sometimes we cannot determine the locations of all contact points, especially where they make contact with the ground, as in the validation case. Another notable issue is that when vehicle-pedestrian contact was only considered, there will be some bias in the optimization results of falling dynamics and the contact position with ground. This issue is critical when the boundary conditions of the MB simulation are utilized in the finite element simulation to determine the degree of landing injury (Wang et al., 2022a). Other accidents, such as two-wheeler accidents, should be reconstructed to further validate the proposed method.

## 5 Limitations and future work

Several limitations must be noted. First, the current study considered only vehicle-pedestrian collisions and did not cover more complex traffic accident scenarios, such as cycling or pushing a bicycle. More impact scenarios need to be included for a more comprehensive investigation. Second, the stiffness of the multi-rigid-body model of the vehicle was set by the stiffness curve of the front parts of similar vehicles, and there is a certain deviation from the stiffness of the real accident vehicle. It will be interesting to include vehicle stiffness as a variable in the optimization in further research. In addition, the friction coefficients were adjusted according to the literature. This study did not consider the friction coefficient as the initial variable of optimization as the objective function determines it. In the present case, the objective function is the Euclidean distances between the known human, vehicle and ground contact points at the time of initial impact. Thus, only the friction coefficient between the car and the pedestrian has an impact on the objective function. However, it is necessary to analyse the effects of other variables in the future. When the objective function is the final located position of a vehicle or pedestrian, the friction coefficients will have an important influence, which leads us to another research topic: how we analyse the effects of different initial variables and different objective functions. Another issue is whether known injuries can serve as an objective function for optimization. The difficulty was how to quantify the specific value corresponding to the real injury in optimization. Third, this study used only a few cases for a preliminary investigation, and additional cases will be needed for more accurate and applicable studies in the future. In this study, the role of the available accident video information in the reconstruction is obvious. Much information such as vehicle collision speed, the range of pedestrian posture and the evaluation of the kinematic process of the reconstructed accident, is obtained from the video information, by which we obtain a more accurate range of initial variables. Notably, cases without available video will be more challenging but also more important, as reconstruction can help establish what actually happened. We need to obtain more accident information to limit the range of optimization initial variables. On the other hand, we need enough deterministic evidence to verify the reconstruction results. In the future, researchers could use limited accident information, such as the correspondence between injuries and traces, the degree of injuries, the angle and distance of initial position and rest position for accident participants, etc., which could be considered objective functions to qualify the final accident reconstruction. In this way, the current methods and multiobjective algorithms could be further tested in traffic accident reconstruction. Of course, under certain conditions, it would be more convincing to use the video information as a test of the final reconstruction results optimized by independent analysts. The basic methodology has now been established, and a wide range of questions need to be addressed in further work. The combined application of MB dynamic and finite element methods would enhance the credibility when simulation results were employed as forensic evidence. The MB dynamic method with an optimization algorithm is used first to extract the initial conditions of the body at the impact with the vehicle, and then the finite element method is used to simulate the injury mechanism.

## 6 Conclusion

In this study, an intelligent approach for the accurate reconstruction of vehicle-pedestrian accidents based on MB dynamic simulation with a multiobjective optimization algorithm was established. 3D laser scanning technology was used to reconstruct a vehicle involved in a traffic accident, and the accident scene was reconstructed using an UAV, combining the pedestrian posture and weather conditions in the video information. Different multiobjective optimization algorithms, such as NSGA-II, NCGA, and MOPSO, influenced the accuracy of the accident reconstruction process. NSGA-II showed better performance and provided a smaller value of the objective function, more alternative solutions and better convergence, than the other two algorithms. The selection and contact of collision marker points during the collision process also had an impact on the accuracy of the accident reconstruction. The larger the number of collision marker points is, the greater the number of alternative solutions that can be obtained. More accurate reconstructions can be obtained when at least one pair of collision markers is set in each of the three phases after a vehicle-pedestrian collision, i.e., when the pedestrian is struck by the vehicle, flips in the air, and falls to the ground. In this paper, three multiobjective optimization methods were used to reconstruct a traffic accident and to more realistically and accurately reproduce the kinematic correspondence between pedestrians and vehicles in road traffic accidents, complementing and refining accident reconstruction optimization methods. The proposed approach showed good potential for improving the identification procedure of vehicle preimpact conditions and the accuracy of accident reconstruction, which can provide supporting evidence for the forensic identification of traffic accidents.

## Data Availability

The datasets generated during and/or analysed during the current study are available from the Dryad Digital Repository: https://doi.org/10.5061/dryad.2ngf1vhs6.
