# Supplementary material for: Multiobjective optimization algorithm for accurate MADYMO reconstruction of vehicle-pedestrian accidents
Source: Front Bioeng Biotechnol. 2022 Dec 5;10:1032621. doi: 10.3389/fbioe.2022.1032621 (PMC9760744; doi:10.3389/fbioe.2022.1032621)
Supplement: Supplementary file 1 [file Table1.DOCX]

Supplementary Material

# Supplementary Figures and Tables (Results related to validation cases)

## Supplementary Table

**Supplementary Table 1** List of the global optimal solution parameters by different multi-objective optimization algorithms in the validation cases

|  | Different algorithms  (I, II) | *V*  (m/s) | *D*  (m) | *α*  (rad) | *β*  (rad) | *γ*  (rad) | *d*(AB)  (m) | *d*(CD)  (m) | *d*(objective)  (m) |
| --- | --- | --- | --- | --- | --- | --- | --- | --- | --- |
| Case 1 | initial value | 14.0000 | 0.3000 | 0.7500 | 0.0000 | -0.1000 | 0.2338 | 0.1950 | 0.4288 |
|  | NSGA-II | 14.1187 | 0.0885 | 0.5367 | -0.1904 | 0.0958 | 0.0024 | 0.0681 | 0.0705 |
|  | NCGA | 14.5413 | 0.0977 | 0.5118 | -0.1467 | -0.1668 | 0.0096 | 0.0631 | 0.0727 |
|  | MOPSO | 13.7452 | 0.0915 | 0.6186 | -0.0928 | 0.0174 | 0.0122 | 0.0651 | 0.0774 |
| Case 2 | initial value | 12.0000 | 0.0000 | 0.5000 | 0.0000 | -0.2000 | 0.0436 | 0.0520 | 0.0956 |
|  | NSGA-II | 12.3362 | 0.1059 | 0.6714 | 0.0358 | -0.1612 | 0.0183 | 0.0165 | 0.0348 |
|  | NCGA | 12.4671 | 0.1019 | 0.6119 | 0.1060 | -0.1494 | 0.0103 | 0.0239 | 0.0343 |
|  | MOPSO | 10.3883 | 0.1362 | 0.6201 | 0.0906 | -0.2431 | 0.0186 | 0.0173 | 0.0359 |

## Supplementary Figures

##
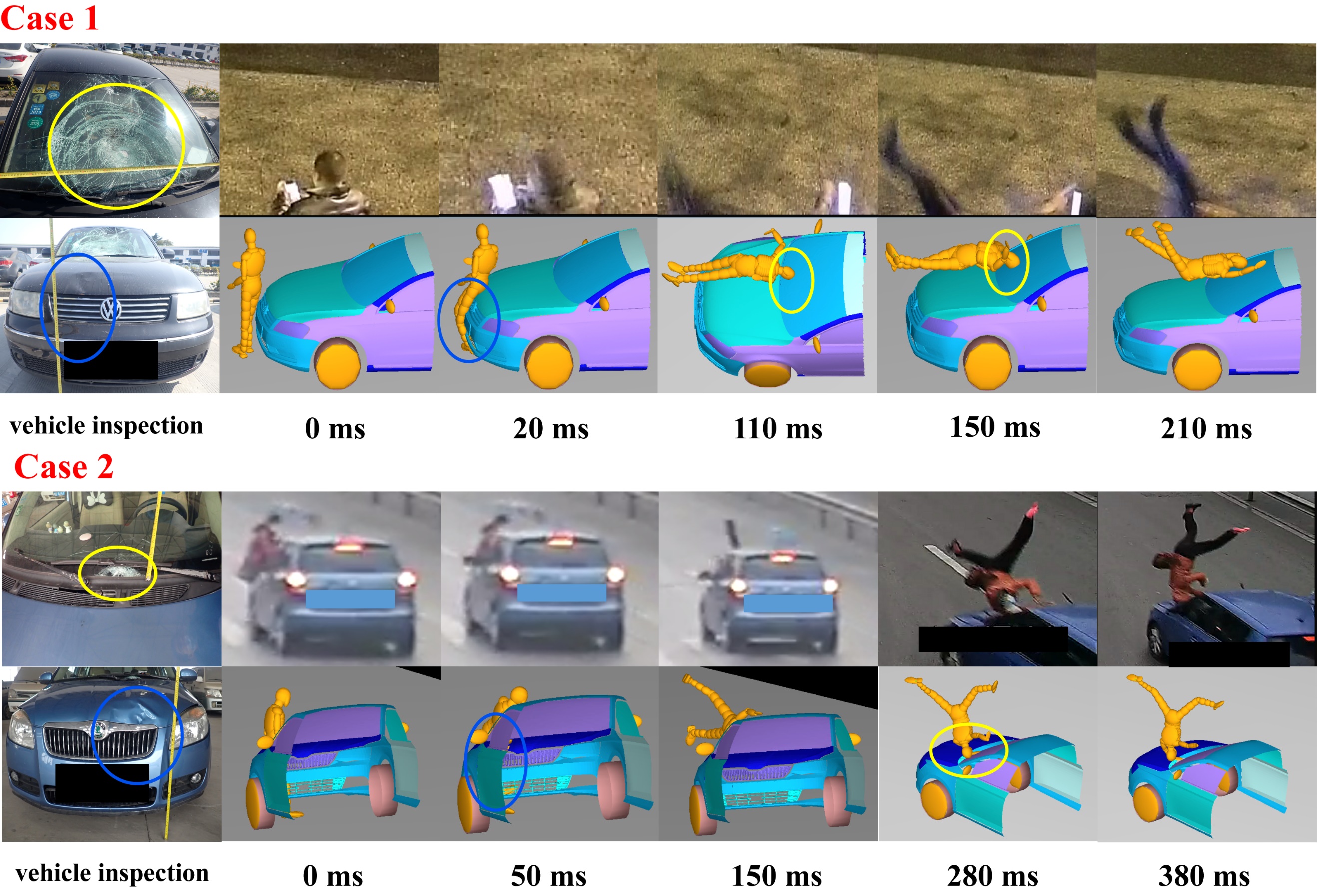


**Supplementary Figure 1.** Comparison of kinematic response between simulation and accident (Informed consent was obtained from the families of the deceased for the relevant content in this figure (Informed consent was obtained from the families of the deceased for the relevant content in this figure.)


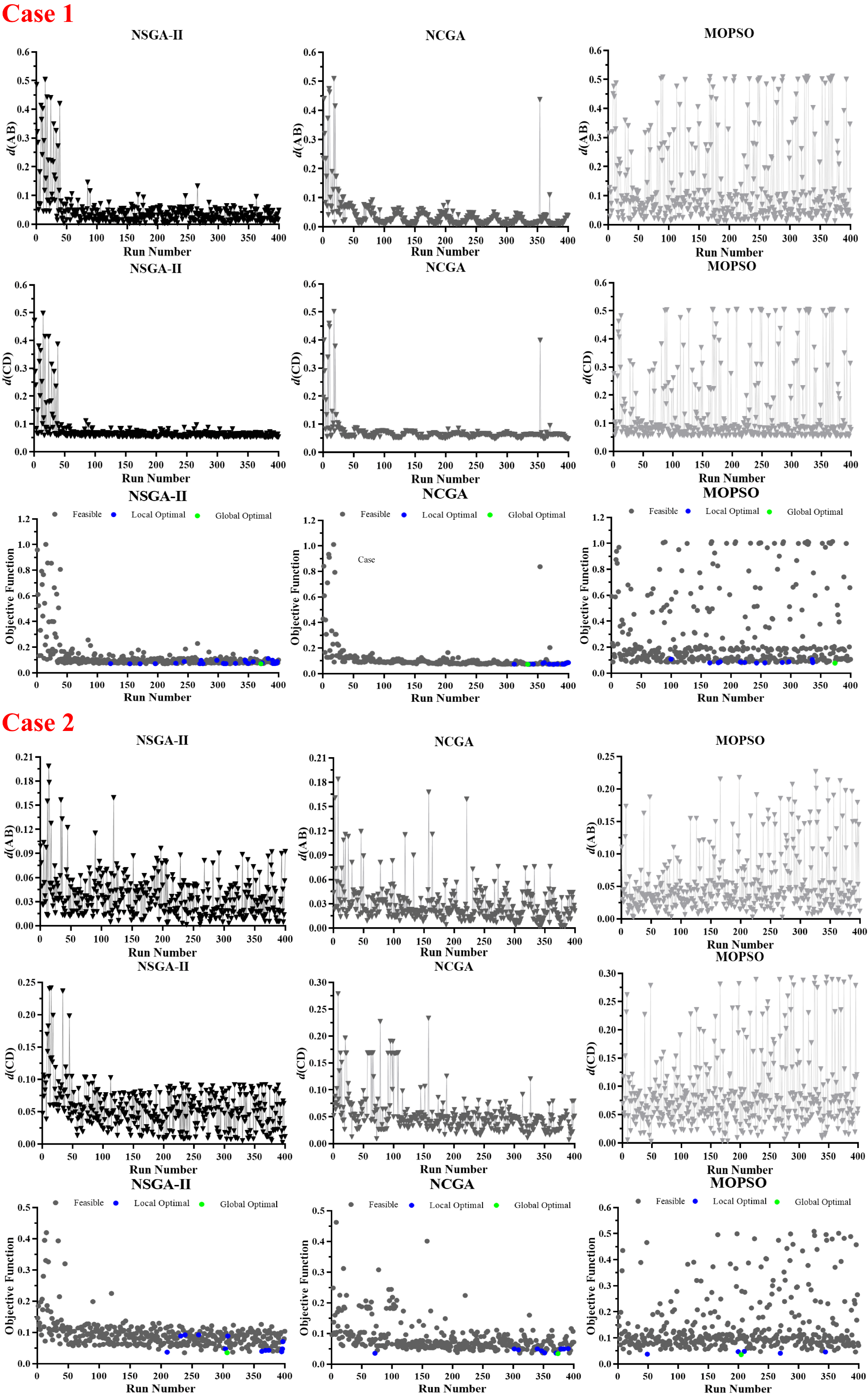


**Supplementary Figure 2.** The simulation histories of different algorithms. The three columns from left to right are NSGA-II, NCGA, MOPSO. The x-axis of each Pareto graph indicates the number of runs, and the y-axis indicates the value of each sub-objective function and the objective function
